# An Angiotensin I-Converting Enzyme Mutation (Y465D) Causes a Dramatic Increase in Blood ACE via Accelerated ACE Shedding

**DOI:** 10.1371/journal.pone.0025952

**Published:** 2011-10-05

**Authors:** Sergei M. Danilov, Kerry Gordon, Andrew B. Nesterovitch, Heinrich Lünsdorf, Zhenlong Chen, Maricela Castellon, Isolda A. Popova, Sergey Kalinin, Emma Mendonca, Pavel A. Petukhov, David E. Schwartz, Richard D. Minshall, Edward D. Sturrock

**Affiliations:** 1 Department of Anesthesiology, University of Illinois at Chicago, Chicago, Illinois, United States of America; 2 Institute for Personalized Medicine, University of Illinois at Chicago, Chicago, Illinois, United States of America; 3 Division of Medical Biochemistry, Institute of Infectious Diseases and Molecular Medicine, University of Cape Town, Cape Town, South Africa; 4 Department of Dermatology, Rush University, Chicago, Illinois, United States of America; 5 Department of Vaccinology and Applied Microbiology, Helmholtz-Center of Infection Research, Braunschweig, Germany; 6 Department of Pharmacology, University of Illinois at Chicago, Chicago, Illinois, United States of America; 7 Chemistry of Life Processes Institute, Northwestern University, Evanston, Illinois, United States of America; 8 Department of Bioengineering, University of Illinois at Chicago, Chicago, Illinois, United States of America; 9 Department of Medicinal Chemistry and Pharmacognosy, College of Pharmacy, University of Illinois at Chicago, Chicago, Illinois, United States of America; Leiden University Medical Center, The Netherlands

## Abstract

**Background:**

Angiotensin I-converting enzyme (ACE) metabolizes a range of peptidic substrates and plays a key role in blood pressure regulation and vascular remodeling. Thus, elevated ACE levels may be associated with an increased risk for different cardiovascular or respiratory diseases. Previously, a striking familial elevation in blood ACE was explained by mutations in the ACE juxtamembrane region that enhanced the cleavage-secretion process. Recently, we found a family whose affected members had a 6-fold increase in blood ACE and a Tyr465Asp (Y465D) substitution, distal to the stalk region, in the N domain of ACE.

**Methodology/Principal Findings:**

HEK and CHO cells expressing mutant (Tyr465Asp) ACE demonstrate a 3- and 8-fold increase, respectively, in the rate of ACE shedding compared to wild-type ACE. Conformational fingerprinting of mutant ACE demonstrated dramatic changes in ACE conformation in several different epitopes of ACE. Cell ELISA carried out on CHO-ACE cells also demonstrated significant changes in local ACE conformation, particularly proximal to the stalk region. However, the cleavage site of the mutant ACE - between Arg1203 and Ser1204 - was the same as that of WT ACE. The Y465D substitution is localized in the interface of the N-domain dimer (from the crystal structure) and abolishes a hydrogen bond between Tyr465 in one monomer and Asp462 in another.

**Conclusions/Significance:**

The Y465D substitution results in dramatic increase in the rate of ACE shedding and is associated with significant local conformational changes in ACE. These changes could result in increased ACE dimerization and accessibility of the stalk region or the entire sACE, thus increasing the rate of cleavage by the putative ACE secretase (sheddase).

## Introduction

Angiotensin I-converting enzyme (ACE, CD143) is a Zn^2+^ dipeptidyl carboxydipeptidase which plays a key role in the regulation of blood pressure and in the development of vascular pathology and remodeling [1]–[4]. ACE is constitutively expressed on the surface of endothelial cells, different absorptive epithelial and neuroepithelial cells [5]–[13], and cells of the immune system (macrophages, dendritic cells) [14]–[16]. Somatic ACE (ACE) contains two catalytic centers in the N- and C-terminal ectodomains [17]. ACE was assigned as a common differentiation marker - CD143 [11], [18].

Besides membrane-bound forms of ACE, blood and other biological fluids contain a variable amount of soluble ACE. Serum ACE most likely originates from endothelial cells [19], mostly from lung – due to preferential ACE expression in lung capillaries [11], [13], [20] - by proteolytic cleavage [21]–[22]. Soluble ACE from CHO cells transfected with human ACE cDNA and porcine ACE have C-termini consistent with cleavage of the Arg1203-Ser1204 peptide bond in a stalk region near the transmembrane domain [23]. The cleavage/secretion process is catalyzed by an unidentified membrane-bound ACE secretase [24]. In healthy individuals, the level of ACE in the blood is very stable [25], whereas granulomatous diseases (sarcoidosis in particular) and Gaucher's disease lead to a significant increase of ACE activity in the blood [26]–[28]. Serial serum ACE measurements now are an essential tool for the diagnosis and monitoring the clinical course of sarcoidosis [29]–[31].

A mutation in the stalk region of ACE – Pro1199Leu [32]–[33] – explained a dramatic (5-fold) increase in ACE activity in the blood of affected individuals from Holland [32], Germany [34], and the USA [35]. Despite the fact that people with this mutation exhibit no clinical abnormalities [32], testing for this mutation is of considerable clinical importance. For example, failure to appreciate that elevation of ACE levels is genetically determined in an affected individual may lead to false diagnosis of neurosarcoidosis and consequently to unnecessary long-term immunosuppressive treatment [34] or diagnostic procedures [36]. Another mutation in the stalk region that introduced a premature termination codon (Trp1197Stop) led to direct secretion of mutant ACE into the circulation and consequently a 14-fold increase in blood ACE [37].

Here we report the identification of a new mutation in ACE, substitution of Tyr in position 465 in the N domain to Asp (Y465D), which is distal to the stalk region, where proteolytic cleavage occurs, and leads to significant increase in the rate of ACE shedding. This mutation occurs in the interface of an N-domain dimer observed during crystallization of the N domain of ACE [38]. Moreover, the Y465D substitution results in significant local conformational changes in ACE. Therefore, it is likely that the Y465D mutation affects the extent of ACE dimerization and increases the accessibility of either the stalk region for cleavage or a secondary binding site/recognition domain of ACE, thus confirming our hypothesis of a link between ACE dimerization and shedding [39]–[40].

## Materials and Methods

### Site-directed mutagenesis and in vitro analysis of the mutant ACEs

cDNAs encoding mutant ACE protein were created by GenScript (Piscataway, NJ) by mutation of the **G**AT codon (Tyr) at position 465 (mature somatic ACE numbering [17]) to codon TAT that encodes an Asp in the expression vector based on pcDNA3.1+/Hygro (Invitrogen Corp., Carlsbad, CA), which contains full-length sACE cDNA controlled by a CMV promoter [41]. Plasmid DNA was sequenced and clones with the desired mutation were selected for each mutation.

Plasmids carrying the cDNA for wild-type (WT) ACE and above mutant were stably expressed in Human Embryonic Kidney (HEK) cells (ATCC, Manassas, VA) using Plus Reagent (Invitrogen Corp., Carlsbad, CA). Culture medium (Ultra-CHO medium, Cambrex Bio-Science, Walkersville, MD or serum-free MEM) from these cells was used as a source of the secreted (soluble) ACE (WT and mutant) for biochemical and immunological characterization. Lysate of these cells obtained with detergent Triton X-100 (0.5% in PBS) was used as a source of membrane-bound form of WT and mutant ACE. Cells at confluence (CHO or HEK) were washed twice with PBS. Triton X-100 (final concentration 0.5% in 50 mM Tris-HCl, pH 7.5 containing 150 mM NaCl) and protease inhibitor cocktail (without EDTA) were added to the cells (1 ml per 60 mm petri dish). After 30 minutes incubation at 4°C, cells were scraped and the whole lysate was centrifuged at 10 000 g to remove cell debris. The supernatant was used as cell lysate. The rate of ACE shedding was determined as the ratio of ACE activity in the culture medium to the sum of ACE activity in the medium and the lysate.

Chinese Hamster Ovary (CHO) cells (ATCC, Manassas, VA) were stably transfected with cDNA coding mutant ACE using the Ca_2_PO_4_ method (Profection Mammalian Transfection System, Promega, Madison, WI). After transfection, cells were grown to confluency in enriched medium [50% Dulbecco's Modified Eagle Medium (DMEM) 50% HAMS-F12 supplemented with 10% fetal calf serum (FCS) and 20 mM HEPES buffer with 400 µg/ml hygromycin (all reagents are from Sigma-Aldrich, St. Louis, MO). Then, cells were lifted and sorted for high expressing cells by flow cytometry, using the N domain-specific anti-ACE mAb 5C5, having the same epitope specificity as mAb i2H5 [42]. Cells were then serum starved overnight in minimal medium [50% DMEM, 50% HAMS-F12, 2% FCS, 20 mM HEPES buffer, 100 U/ml penicillin and 100 µg/ml streptomycin], and the medium assayed for ACE activity.

### ACE activity assay

ACE activity in culture medium (soluble ACE) or lysates of ACE-expressing cells (membrane-bound form) was measured using a fluorimetric assay with two ACE substrates - 2 mM Z-Phe-His-Leu [43] or 5 mM Hip-His-Leu [44]. Briefly, 20–40 µl aliquots of culture medium or lysates (diluted 1/5 in PBS-BSA (0.1 mg/ml), were added to 200 µl of ACE substrate and incubated for the appropriate time at 37°C. The His-Leu product was quantified fluorimetrically.

### Western blot analysis of mutant ACEs

Samples from HEK cells expressing sACE for SDS electrophoresis were equilibrated to a final ACE activity of 5 mU/ml (Hip-His-Leu as a substrate) and were run using 7.5% Tris-HCl pre-cast SDS-PAGE gels (Bio-Red Laboratories, Hercules, CA). After electrophoretic transfer of proteins to microporous PVDF-Plus membranes, each membrane was incubated in 10 mM Tris-HCl (pH 8.0) buffer containing 150 mM NaCl, 0.05% Tween 20, and 5% dry milk prior to incubation overnight at 4°C with mouse mAbs to sequential epitopes on human ACE, suitable for detection of the denatured ACE - 3C5, 1D8, 5C8 [45]–[47]. Subsequent steps were carried out with the biotin/streptavidin system (Amresco, Solon, OH) and peroxidase activity was developed using WestPico Super Signal Chemiluminescense substrate (Pierce, Rockford, IL).

Samples from CHO cells expressing sACE were separated by SDS-PAGE using the Mini PROTEAN™ III system (BIO-RAD, Hercules, CA) with 6% resolving and 3% stacking gels in a Tris-glycine tank buffer (pH 8.3). Protein was transferred to Hybond ECL nitrocellulose membrane (GE Healthcare LifeSciences, Buckinghamshire, UK) in transfer buffer (20 mM Tris-base, 150 mM glycine, 20% methanol) using the Mini PROTEANTM III system. For detection of protein, membranes were incubated in blocking buffer (Tris-buffered saline, 0.1% tween-20, 5% skim-milk) containing culture fluid of the anti-ACE mAb 1D8, specific to C domain [46]–[47]. Binding of mAb was detected using goat anti-mouse HRP-conjugated antibody using the Immun star WesternC™ detection system (BIO-RAD, Hercules, CA). Chemiluminescence was detected with a chemiluminescence detector (G:BOX Chemi, Syngene, Frederick, MD). Densitometry of detected protein was performed using GeneTools software (Syngene, Frederick, MD).

### Immunological characterization of the mutant ACE (Plate immunoprecipitation assay)

96-well plates (Corning, Corning, NY) were coated with anti-ACE mAbs via goat anti-mouse IgG (Pierce, Rockford, IL) bridge [42] and incubated with samples of lysates from ACE- expressing cells, representing membrane-bound form of ACE or culture medium from these cells, representing soluble ACE secreted from these cells, which were equilibrated for ACE activity with Hip-His-Leu as a substrate. After washing of unbound ACE, plate-bound ACE activity was measured by adding a substrate for ACE (Hip-His-Leu) directly into wells [42].

### Cell ELISA

Stable CHO-sACE expressing cell lines (sACE-A10) were grown to confluency overnight in microtitre plates in enriched medium. Once confluent, cells were incubated on ice with blocking buffer (PBS with 2% skim milk) and then in the presence of 10 µg/ml anti-ACE mAbs in the same buffer. Cells were fixed with 4% paraformaldehyde and incubated in blocking buffer containing a goat anti-mouse IgG HRP-conjugated antibody, which was used to detect the amount of mAb bound to ACE spectrophotometrically using a tetramethylbenzidine (TMB) substrate.

### MALDI-TOF MS analysis of mutant ACE-Y465D

Purified ACE-Y465D was resolved by SDS-PAGE and protein detected by Coomassie stain. The sACE band was excised, cut into 1 mm^2^ pieces and destained with 200 mM NH_4_CO_3_:acetonitrile (ACN) (50∶50) until clear. Samples were dehydrated with 100% ACN and dried on a Savant SpeedyVac (ThermoScientific, Farmingdale, NY). Samples were sent to the Centre for Proteomic and Genomic Research (CPGR, Cape Town, South Africa) for further analysis. Briefly, samples were reduced with 5 mM Tris (2-carboxyethyl) phosphine (TCEP) (Fluka-USA, Milwaukee, WI) in 100 mM NH_4_CO_3_ in the dark for 30 minutes at room temperature. Excess TCEP was removed and the gel slices dehydrated. Cysteine protection was performed by carbamidomethylation with 100 mM iodoacetamide (Sigma-Aldrich, St. Louis, MO) in 100 mM NH_4_CO_3_ for 30 minutes at room temperature in the dark. The gel slices were then dehydrated, washed with 50 mM NH_4_CO_3_ and dehydrated. An in-gel tryptic digest was performed by rehydrating the gel slices in trypsin solution (Promega, Madison, WI) to a 20 ng/µl final concentration and incubating at 37°C overnight. Peptides were extracted from the gel slices with 50 µl 0.1% trifluoroacetic acid (TFA) (Sigma -Aldrich, St. Louis, MO). Samples were dried down, resuspended in 50 µl dH_2_O and further dried down to 20 µl to remove residual NH_4_CO_3_. Peptides were spotted onto a 10 mg/ml α-cyano-4-hydroxycinmanic acid matrix (Fluka-USA, Milwaukee, WI) in 80% ACN, 0.2% TFA for a final concentration of 5 mg/ml matrix in 40% ACN, 0.1% TFA, 10 mM NH_4_H_2_PO_4_. Mass spectrometry was performed with a 4800 MALDI TOF/TOF (Applied Biosystems, Foster City, CA) with all spectra recorded in positive reflector mode. Spectra were generated with 400 laser shots/spectrum at a laser intensity of 3800 (arbitrary units) with a grid voltage of 16 kV. Peptides spots were internally calibrated with trypsin autolytic fragments.

### Molecular modeling of ACE

Analysis of the N domain was based on the recently published crystal structure [38] (PDB accession number 3NXQ) and the C domain was based on the structure resolved by Natesh *et al*. [48] (PDB accession number 1O86). PYMOL (http://www.pymol.org) was used to make the Y465D substitution *in silico* using the in-built mutagenesis software. A model of complete human sACE was constructed using the model of porcine sACE based on electron microscopy (EM) [49] and visualized using Chimera [50]. Arrangement of X-ray 3D densities of the N and C domains (PDB-IDs: 3NXQ_chain A and 1O86) into the porcine somatic lung ACE electron microscope model was done (1) by alignment of both domains' N-termini to north and their C-termini accordingly to south, along the longitudinal axis of the ACE-model, (2) by setting the domains' X-ray 3D densities to 2,3 nm resolution and fitting them into the ACE-model semi-automatically with the ‘Fit in map’ module, and (3) by shifting and rotating both domains manually to re-align them to the models longitudinal axis and to get the N-domain Glu590 residue proximal to the C-domain's N-terminus, giving it the optimal fit for the ‘hinge’ residues Val591 to Gly610. The epitopes were marked on the N and C domains according to [40], [51]–[55] and two such ACE 3D models were arranged back-to-back with both their N-domains, similar to the dimer of human ACE (3NXQ). Structural figures were generated in Chimera or PYMOL. Protein-protein interactions were identified using PDBSum [http://www.ebi.ac.uk/pdbsum/]. Analysis of the effect of the mutation on residues involved in dimer association was performed using PISA [56].

## Results and Discussion

### Identification of the novel ACE mutation

Certain individuals within a family demonstrated plasma ACE levels 5- to 7-fold higher than normal (Nesterovitch et al. 2011, in preparation). A heterozygous point mutation was identified in exon 8 of the ACE gene, which is a T>G transition that converts codon TAT (encoding Tyr at position 465 - mature somatic ACE numbering [17]) to **G**AT (encoding Asp). No other mutation was present in ACE gene exons from these subjects, suggesting a possible link between the high plasma ACE observed and the Y465D mutation. Some of the carriers of this mutation demonstrated symptoms of nausea, vomiting, fatigue, or depression. These might be caused by changes in ACE hydrolysis of neuropeptides (ACE substrates) or by ACE signaling (for example, via changes in ACE dimerization) induced by ACE substrates or endogenous ACE inhibitors; however, the etiology remains unclear.

The regulation of ACE shedding by the putative secretase is likely to differ significantly in various cell types. ACE expression levels are comparable in lung endothelial cells and in the epithelial cells of the proximal tubules of the kidney and seminiferous tubules [11], [13], [57]. However, the respective levels of soluble ACE in these three compartments differ by 2500-fold, being highest in seminal fluid, 50-fold less in the blood and further 50-fold decrease-in the urine (data not shown). Thus, determining the effect of the Y465D mutation on shedding and/or dimerization in different cell lines could be more informative.

The elevated plasma ACE levels observed with the Y465D substitution are likely due to: a) increased ACE expression in cells or b) increased ectodomain shedding of ACE. To elucidate the mechanism(s) involved in the elevation of plasma ACE in the affected individuals, we performed site-directed mutagenesis of human recombinant ACE, expressed this mutant in HEK and CHO cells, and compared the biochemical and immunological characteristics of this mutant with wild-type (WT) ACE.

### Expression of Y465D in HEK and CHO cells

**Figure 1:**
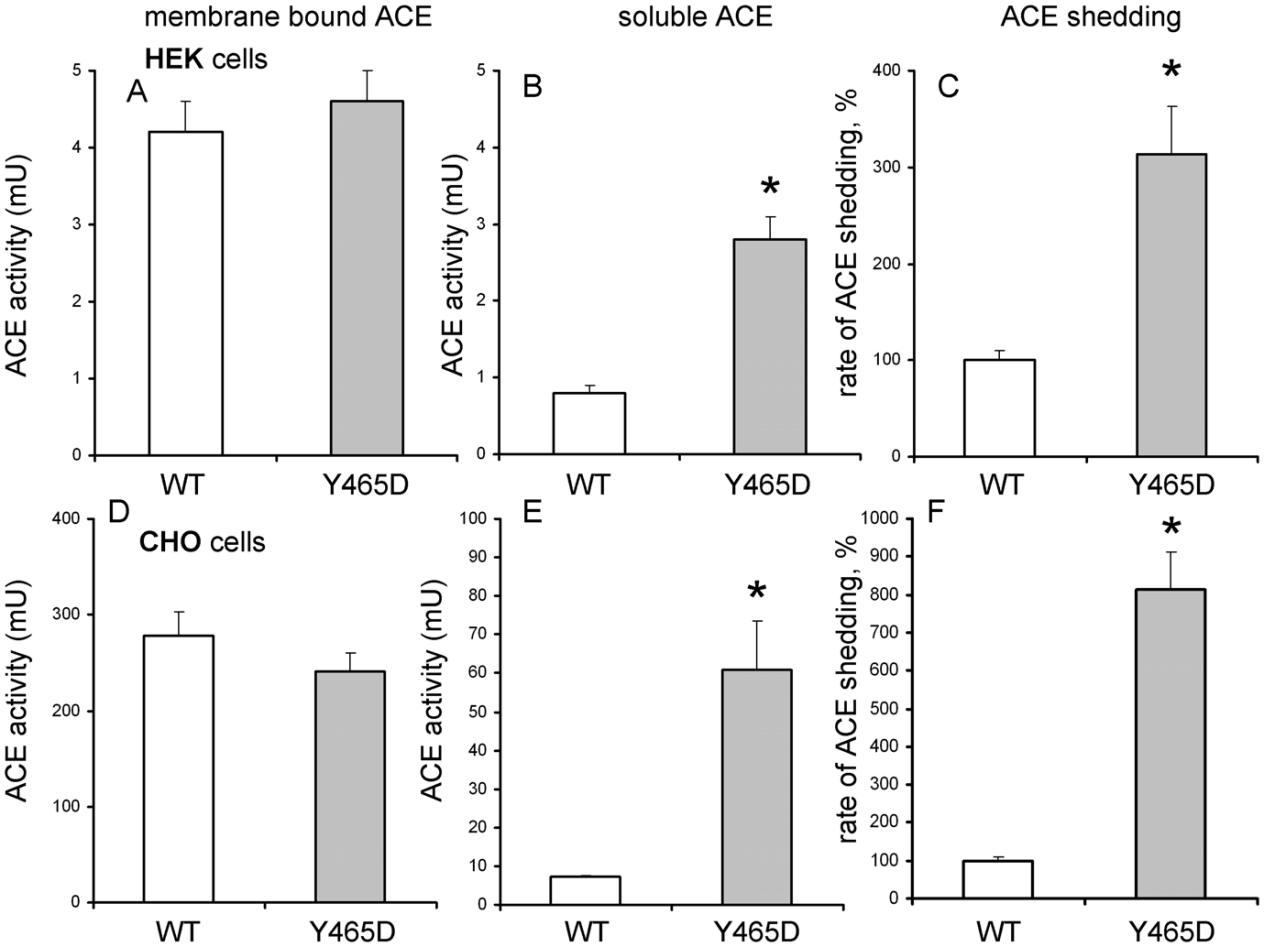
The rate of mutant ACE shedding. **A,D.** Cell-associated ACE activity. Lysates from thoroughly washed cell monolayers from 35 mm culture dishes were prepared using Triton X-100 as detergent. Membrane-bound ACE activity was determined fluorimetrically with Z-Phe-His-Leu as a substrate and expressed as the total mU in each sample and is the mean+SD of four experiments performed in du- or triplicates. **B,E.** Soluble (secreted) ACE activity. The ACE activity in the culture medium of ACE expressing cells from which the cell pellets were used in part A, D was determined fluorimetrically as above. **C,F.** Rate of ACE secretion. The rate of ACE cleavage was determined as the ratio between ACE in the culture medium and the sum of cell-associated and secreted ACE. Data are expressed as a percentage from the mean of WT ACE.

After stable expression of the Y465D mutant ACE (ACE-Y465D) in HEK and CHO cells, we determined the enzymatic activity of the soluble and membrane-bound forms, the rate of shedding, and possible structural abnormalities of ACE-Y465D compared to WT. ACE activity of the membrane-bound form of ACE-Y465D was comparable with WT ACE. However, ACE-Y465D activity in the culture medium and thus its rate of shedding was 3- and 8-fold higher than WT in HEK and CHO cells, respectively (Fig. 1). This occurred regardless of whether artificial tripeptide substrates (HHL or ZPHL) or longer natural substrates (substance P – data not shown) were used. Moreover, we also tested the amount of immunoreactive ACE protein in the culture medium using a plate immunoprecipitation assay [58]. For the Y465D mutant, the amount of ACE protein and activity increased to a similar extent (not shown). We also demonstrated that the inhibition of mutant ACE by enalaprilat was similar to WT ACE (not shown).

**Figure 2:**
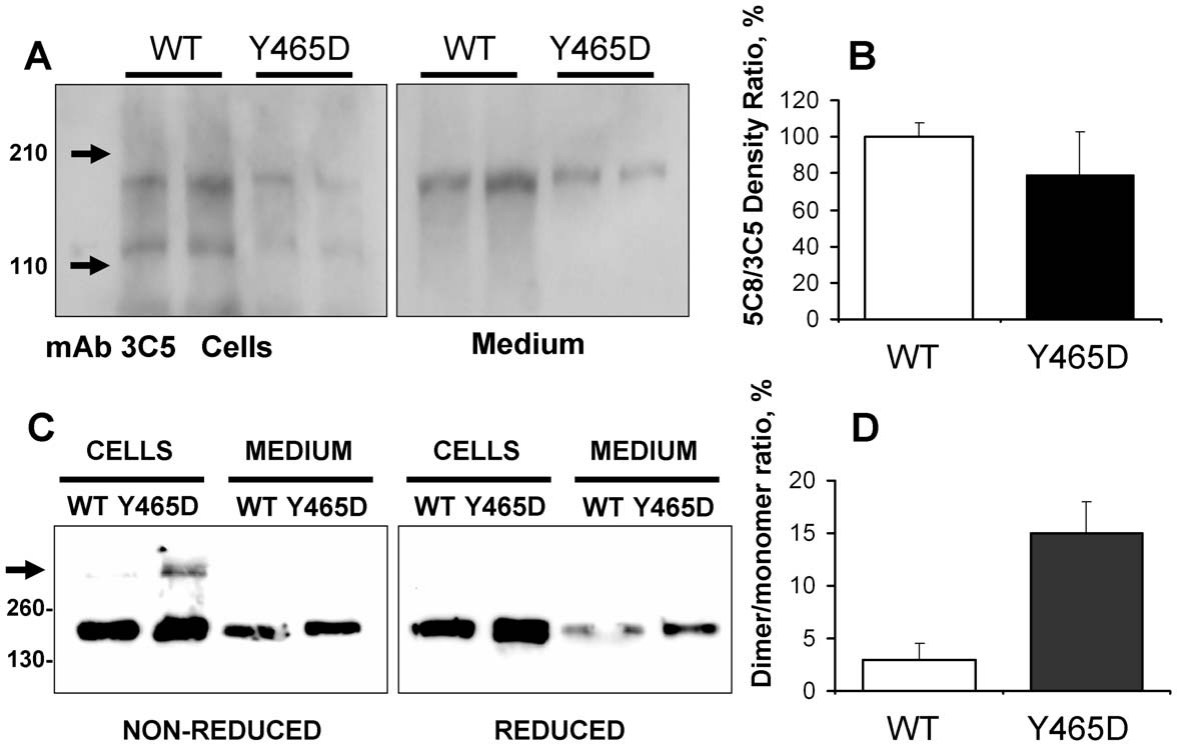
Western blot analysis of normal and mutant ACEs. **A.** The lysates and culture medium of HEK cells expressing WT and mutant (Y465D) ACE (normalized by equal ACE activity loading −5 mU/ml) were subjected to SDS-PAGE (7.5% gel) in reducing conditions for Western blotting with mAbs 3C5 and 5C8 recognizing different sequential epitopes on the C domain of ACE [45]–[47]. Proteins transferred on PVDF-Plus membrane were revealed with 2 µg/ml of indicated mAb. Molecular weight markers are shown by arrows on the left of panel A, which is a representative experiment. **B.** Revelation of WT and mutant ACE presented in panel A (with mAb 3C5) and with mAb 5C8 (not shown) was quantified by densitometry of the bands. Data presented as a ratio of density with mAb 5C8 to that with mAb 3C5 by the image analysis (densitometry) using ImageJ software (NIH). Data are expressed as mean ± SD of 3 independent experiments. **C.** Western blot analysis was performed on total cell lysate and concentrated medium from CHO cells transfected with WT and ACE-Y465D. Samples were separated by 6% SDS-PAGE in the presence (*right*) or absence (*left*) of 2-mercaptoethanol. Immunoblotting was performed and ACE was detected with the C domain-specific mAb 1D8 [47]. **D.** Densitometric analysis of cell lysates separated by Western blot, calculated as the percentage dimer of monomer (D/M). Data is the mean ± SD of samples prepared in duplicate.

Western blot analysis was performed on total cell lysate and medium harvested from HEK and CHO cells expressing WT and ACE-Y465D to confirm that the mutation did not alter ACE expression. The cell-associated WT and mutant ACE resolved at approximately 180 kDa (Fig. 2A, C). The lower molecular weight band in cell lysate likely represents a non-specific band as it was observed in most lysates with different anti-ACE mAbs. A higher molecular weight form >300 kDa was observed for both WT and the ACE-Y465D [seen only in CHO cells expressing Y465D (Fig. 2C,D) likely due to enrichment of ACE by flow cytometry] consistent with a disulphide-mediated ACE dimer [39], [55], [59], since this form is not observed in the presence of reducing agent. Densitometric analysis of cell lysate indicated a 5-fold increase in the ratio of dimer to monomer with ACE-Y465D (Fig. 2D), but the formation of dimers was not consistently observed. This reflects the limitations of determining dimer formation using SDS-PAGE [59] and requires verification by other approaches. In both cell associated and soluble forms, the mutant resolves slightly higher than the WT, which could be the result of alterations in post-translational processing such as glycosylation or phosphorylation due to accelerated shedding and/or accelerated trafficking of ACE-Y465D to the cell membrane. These results suggest that the mutation has no aberrant effect on ACE expression. Therefore, the elevated plasma ACE levels observed in individuals expressing the Y465D mutation are more likely to be the result of changes in the ectodomain shedding than the result of increased protein expression.

### Immunological characterization of the ACE-Y465D

The 5-fold increase in shedding of the Pro1199Leu mutation [32] demonstrates the role of juxtamembrane residues in the recognition of the ACE sheddase. In this light, the dramatic effect of the N domain Y465D mutation, distal to the stalk region, on ectodomain shedding is intriguing. Moreover, the binding of two mAbs (9B9 and 3A5) to overlapping epitopes on the N domain of ACE results in a 2- to 4-fold increase in ACE shedding [60]. Therefore, conformational changes in the N domain of ACE, induced by mAb binding, may lead to changes in conformation of the stalk region or exposure of a sheddase recognition domain in ACE distal to the stalk cleavage site [40], [51], [55].

**Figure 3:**
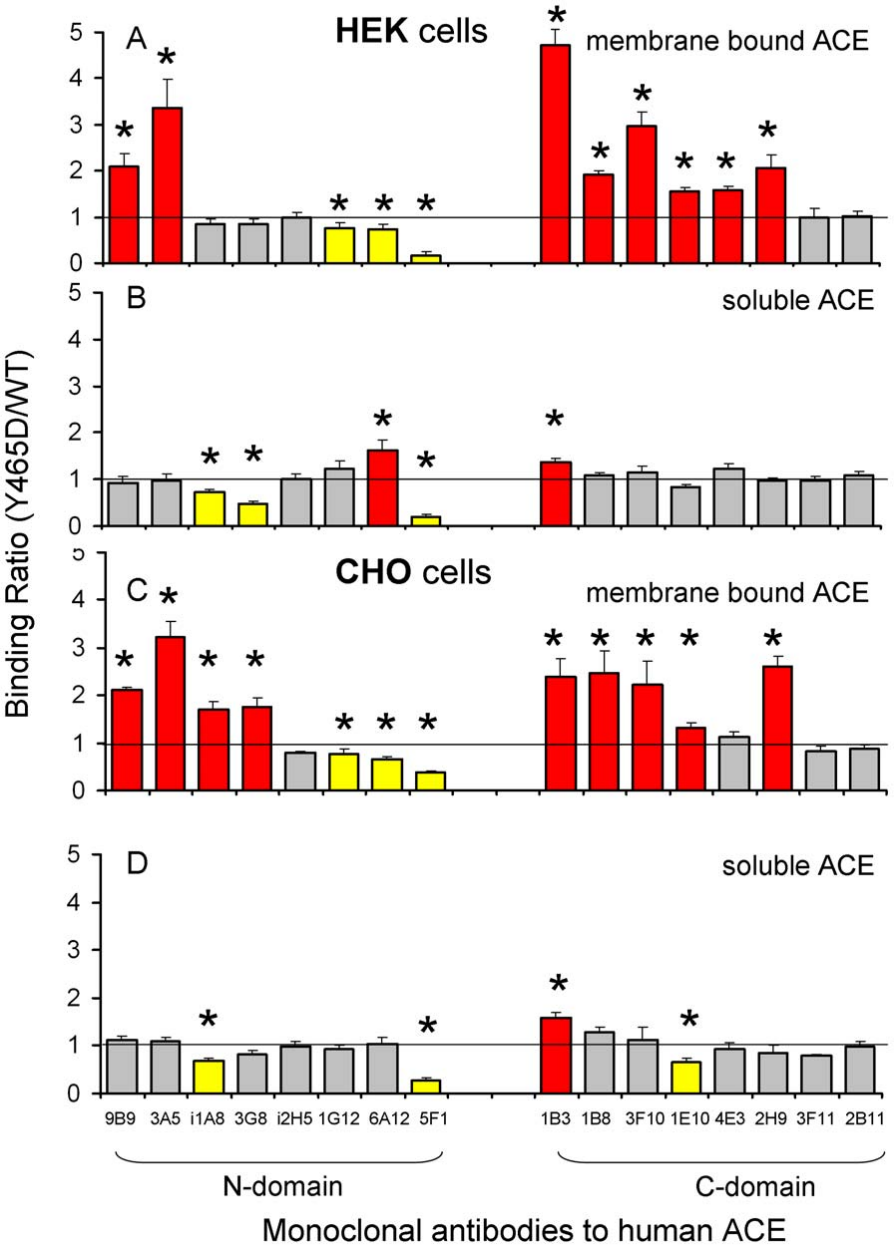
Conformational fingerprint of mutant ACE. **A–D.** Precipitation of ACE activity from cell lysates (membrane-bound form) and culture medium (soluble form) from HEK and CHO cells expressing WT and mutant (Y465D) ACE was estimated using 16 mAbs to the epitopes localized on the N and C domains of human ACE. Samples were equilibrated according to 5 mU/ml of the ACE activity with Hip-His-Leu as a substrate and incubated with a wells on the microtiter plate covered by mAbs to ACE via goat-anti-mouse IgG; then precipitated ACE activity was quantified by fluorimetric assay - plate precipitation assay [42]. Data are expressed as a ratio of ACE activity precipitated by different mAbs from mutant ACE to that of WT ACE. Data presented as a mean of 3–4 independent determinations in duplicate. * p<0.05 indicates ratio shown is significantly different from 1. mAbs showing significant changes in binding compared to WT are colored (red: increase >20%, yellow: decrease >20%).

To investigate the conformational changes induced by the Y465D mutation, we performed conformational fingerprinting of ACE using 16 mAbs to different epitopes on the N and C domains [61]. Of particular interest were the mAbs whose interactions with ACE are known to affect shedding, namely the N-domain specific mAbs 9B9, 3G8 and 3A5 [40], [51], [55], [60], and those whose epitopes were occluded by the opposite domain. These include the epitopes of mAbs 1G12 and 6A12 in the N domain [52] and mAbs 1E10, 2H9 and 4E3 in the C domain [54]. Multiple conformational changes in the ACE molecule were observed as a result of this single mutation (Fig. 3). Unlike previous single amino acid mutations which only affected mAb binding to the specific epitope where the mutation occurs, Y465D appears to affect binding of 12 mAbs to distal epitopes (Fig. 3A and C). Interestingly, the changes in mAbs binding were much more prominent for the membrane-bound form than for the soluble form of ACE (Fig. 3). We demonstrated previously, by determining the conformational fingerprint of ACE, that the ACE conformation differed significantly between the soluble and membrane bound forms [61]. The native conformation of ACE on the cell surface, determined by cell ELISA, also differed significantly from soluble form. In particular, the epitopes for mAbs 1B3, 1B8/3F10, and 1E10 were not exposed on native, catalytically active ACE expressed on the surface of CHO cells [54]. Moreover, the kinetic characteristics, including the domain specificity of some substrates, differed significantly between solubilized ACE and ACE expressed on the cell surface [62].

**Figure 4:**
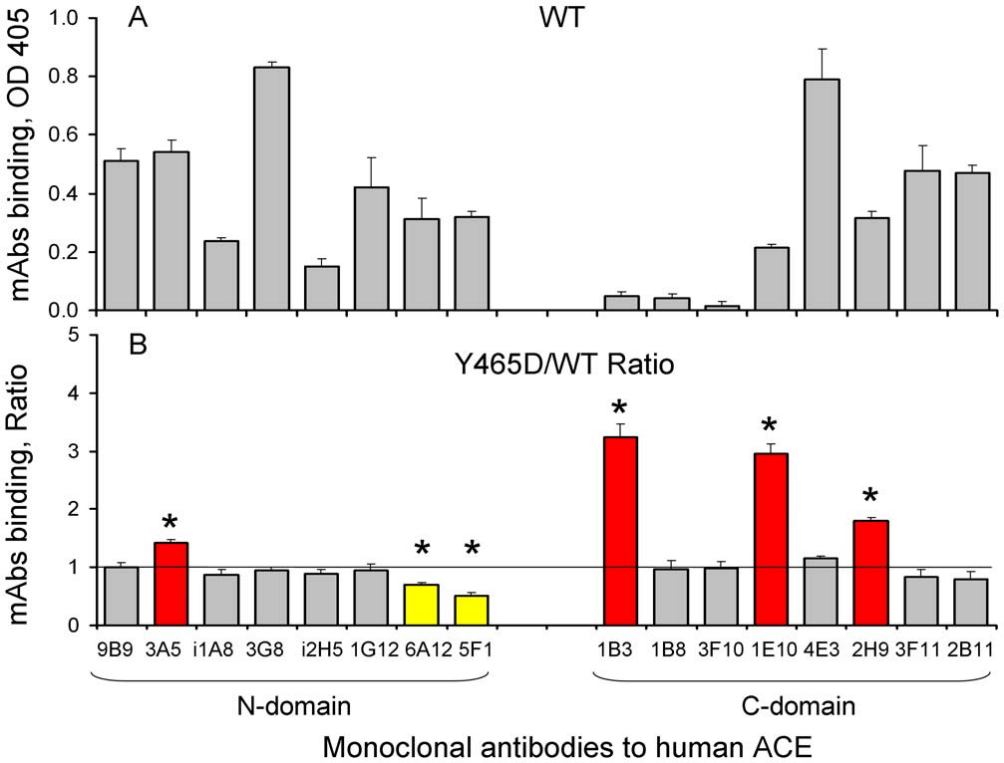
Binding of mAbs to mutant ACE on the surface of CHO cells (Cell ELISA). Cell ELISA assay was performed on CHO cells expressing WT and mutant (Y465D) ACE using the panel of 16 mAbs directed to different epitopes on the N- and C domains of human ACE. **A.** Binding of mAbs to WT ACE determined via secondary Ab - anti-mouse IgG conjugated to peroxidase - optical density at 405 nm. Non-specific binding of the secondary mAbs to control non-immune mouse IgG was subtracted. **B.** Data are expressed as a percentage of mAbs binding to surface of CHO cells expressing mutant ACE to that expressing WT ACE and normalized for surface ACE expression. Data presented as a mean of 3–4 independent determinations in duplicate. mAbs showing significant changes in binding compared to WT are colored (red- increase >20%, yellow-decrease >20%). * p<0.05 indicates ratio shown is significantly different from 1.

Cell ELISA using the panel of domain-specific anti-ACE mAbs was performed on mutant and WT ACE expressed on the surface of CHO cells (Fig. 4). Stable CHO-ACE cell lines (WT and Y465D) were subjected to flow cytometry sorting to increase ACE expression, and thus, to increase the sensitivity of the cell ELISA. Binding of these 16 mAbs to surface WT ACE differs by more than an order of magnitude, reflecting differences in affinity as well as in exposure of the ACE epitopes for different mAbs (Fig. 4A). The Y465D substitution significantly increased binding of 4 mAbs (3A5, 1B3, 1E10 and 2H9) and decreased binding of mAb 5F1 and 6A12 (Fig. 4B).

**Figure 5:**
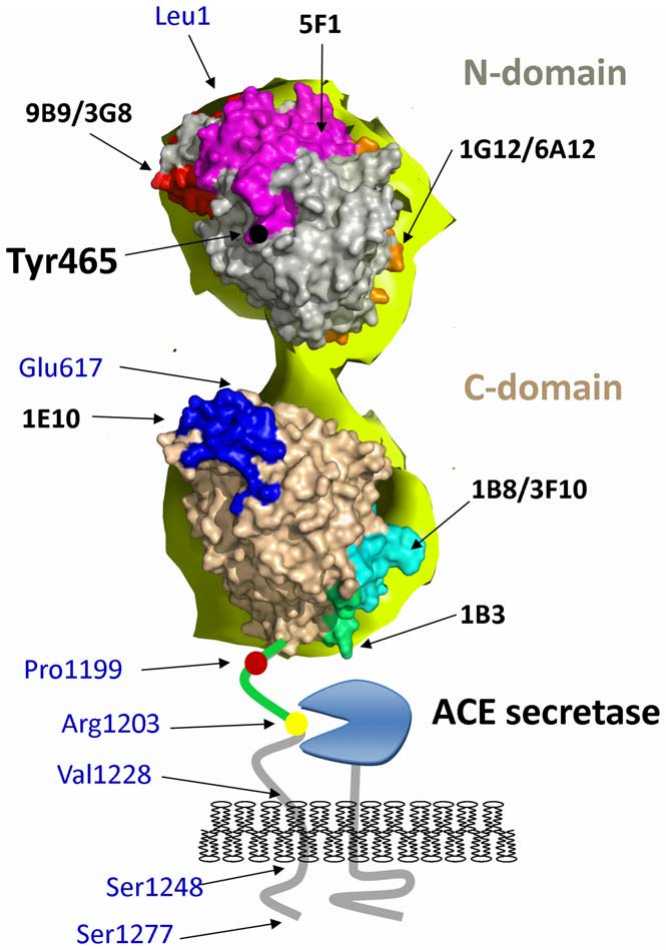
Epitope mapping of human sACE using EM-based model. Model of human sACE was generated using an EM-based model of porcine sACE [49]. Epitopes localization (colored spots) were taken from the following publications [40], [51]–[55].. Relevant amino acid residues in the ectodomain and juxtamembrane region of ACE and a cartoon of the membrane-bound ACE secretase are indicated: Leu1 and Asp616-N terminal residues of the N- and C-domains, respectively; Pro1193 –the last amino acid residues seen in the 3D structure of C domain; Pro1199 – localization of P1199L mutation causing 5-fold increase in blood ACE levels [32]; Arg1203-C-terminal amino acid residue of soluble ACE [23]; Val1228-Ser1248-transmembrane domain [65]; Ser1277-C-terminal amino acid residue of full-length somatic ACE [17].

There was a consistent loss of affinity for the N-domain specific mAb 5F1 with cell-associated and soluble mutant ACE in the precipitation assay (Fig. 3) as well as membrane-bound ACE-Y465D in the cell ELISA (Fig. 4B). Y465 might participate directly in mAb 5F1 binding to ACE, because it is localized close to/within the epitope for mAb 5F1 [53] (Fig. 5), or the mutation has resulted in gross conformational changes that have altered the mAb 5F1 epitope. Alternatively, the changes in putative ACE dimerization have masked the epitope for mAb 5F1.

Similarly, a consistent increase in affinity for the C-domain specific mAb 1B3 was observed across these experiments, the epitope of which lies in the C-terminal region of the C domain, including the stalk region (Fig. 5) [46], [54]. It has been noted that mAb 1B3 binding indicates rearrangement of the juxtamembrane region [63] and an increase in binding of mAb 1B3 is consistent with increased shedding. Rearrangement of the stalk region and exposure of the cleavage site is most likely the cause of both the increase in affinity for mAb 1B3, and/or greater accessibility of the secretase, resulting in increased shedding.

Therefore, the alterations in the conformational fingerprint of ACE, exposing some epitopes (9B9, 3A5, 1B3, 1B8/3F10, 1E10/4E3, 2H9) and masking others (1G12/6A12, 5F1) were revealed by the panel of domain-specific mAbs. These alterations, induced by a single amino-acid mutation in the N domain, indicate conformational changes of the ACE molecule, which might be due to changes in the extent of ACE dimerization.

### Induction and inhibition of mutant ACE shedding

**Figure 6:**
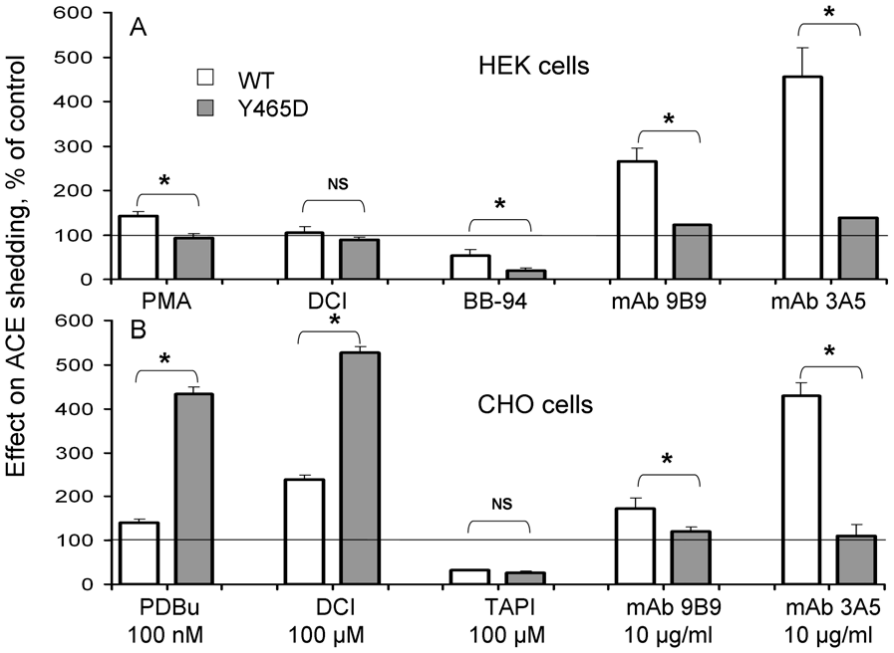
Stimulation and inhibition of mutant (Y465D) shedding. **A.** HEK cells expressing WT and mutant (Y465D) ACE were incubated in serum-free basal medium containing phorbol-12-myristate-13-acetate (PMA), 3,4-dichloroisocoumarin (DCI), the hydroxamic acid-based matrix metalloprotease inhibitor, batimastat (BB-94) or with ACE mAbs (9B9 or 3A5) for 24 hours. **B.** CHO cells expressing WT and mutant (Y465D) ACE were incubated in minimal medium containing phorbol 12,13-dibutyrate (PDBu), DCI, tumour necrosis factor-α protease inhibitor (TAPI) or with ACE mAbs (9B9 or 3A5) for four hours. Shedding was calculated as described in Figure 1. Data is the mean ± SD of two experiments performed in duplicate. A two-way ANOVA was performed for untreated versus treated for each cell line and indicated as * p<0.01.

In order to characterize the mechanism of mutant ACE shedding further we tested known inducers and inhibitors of WT ACE shedding on CHO and HEK cells expressing ACE-Y465D. The effect of both stimulation of shedding by the phorbol esters, PMA (HEK) or PDBu (CHO), and the serine protease inhibitor DCI, as well as inhibition by hydroxamate-based inhibitors of matrix metalloproteases (batimastat- BB-94 and TAPI) was determined (Fig. 6). Phorbol ester induced basal shedding of ACE-Y465D in CHO cells by approximately 4.5-fold, while DCI caused a 5-fold increase in shedding in CHO cells (Fig. 6B) and had no effect on ACE shedding in HEK cells (Fig. 6A). Shedding was significantly inhibited in the presence of batimastat in HEK cells and by TAPI in CHO cells. The different shedding profiles between the two cell types expressing mutant ACE indicates that our understanding of the complex mechanism of ACE shedding is still incomplete. Furthermore, the regulation of ACE shedding likely includes other putative ACE- or ACE secretase-binding proteins whose expression in CHO and HEK cells could be very different.

We also tested the ability of mAbs, known to induce ACE shedding in WT ACE (mAbs 9B9 and 3A5 [40], [51], [55], [60] on the shedding of mutant ACE, expressed in HEK and CHO cells (Fig. 6). The Y465D substitution abolished the mAb-induced shedding observed with WT ACE on the surface of CHO and HEK cells. This could mean that mutant ACE has already formed the conformation sACE adopts upon binding of mAb 3A5, and mAb 9B9 to a lesser extent, and thus re-sets the basal levels of shedding by allowing better access of the secretase.

These results support the hypothesis that a mutation in the N domain of ACE can induce conformational changes that increase access of the ACE secretase, either through exposure of the stalk region or of a recognition domain. This is indicated by the increased affinity of C-domain specific mAbs 1E10 and 2H9 for the membrane-bound form of mutant ACE (Fig. 4B). The epitopes for these mAbs are located on the N-terminal region of the C domain (Fig. 5) and previous findings indicate that they are occluded by the N domain [54] and may form a conformation similar to the compact structure proposed previously [64]. Increased interaction of these mAbs with mutant ACE (Y465D) indicates that this region has become exposed, allowing for better access of the mAbs, most likely due to movement of the N domain as the protein moves into a more extended conformation. This is further supported by the fact that regions distal to the proposed domain interaction surface also seem to have undergone changes since the anti-N domain mAbs 9B9, 3A5, 3G8 and i1A8 on CHO cells bind the cell-associated mutant ACE with a higher affinity and mAbs 9B9 and 3A5 on HEK cells bind soluble and membrane-bound mutant ACE (mAb 3A5) better.

### Cleavage site determination

**Figure 7:**
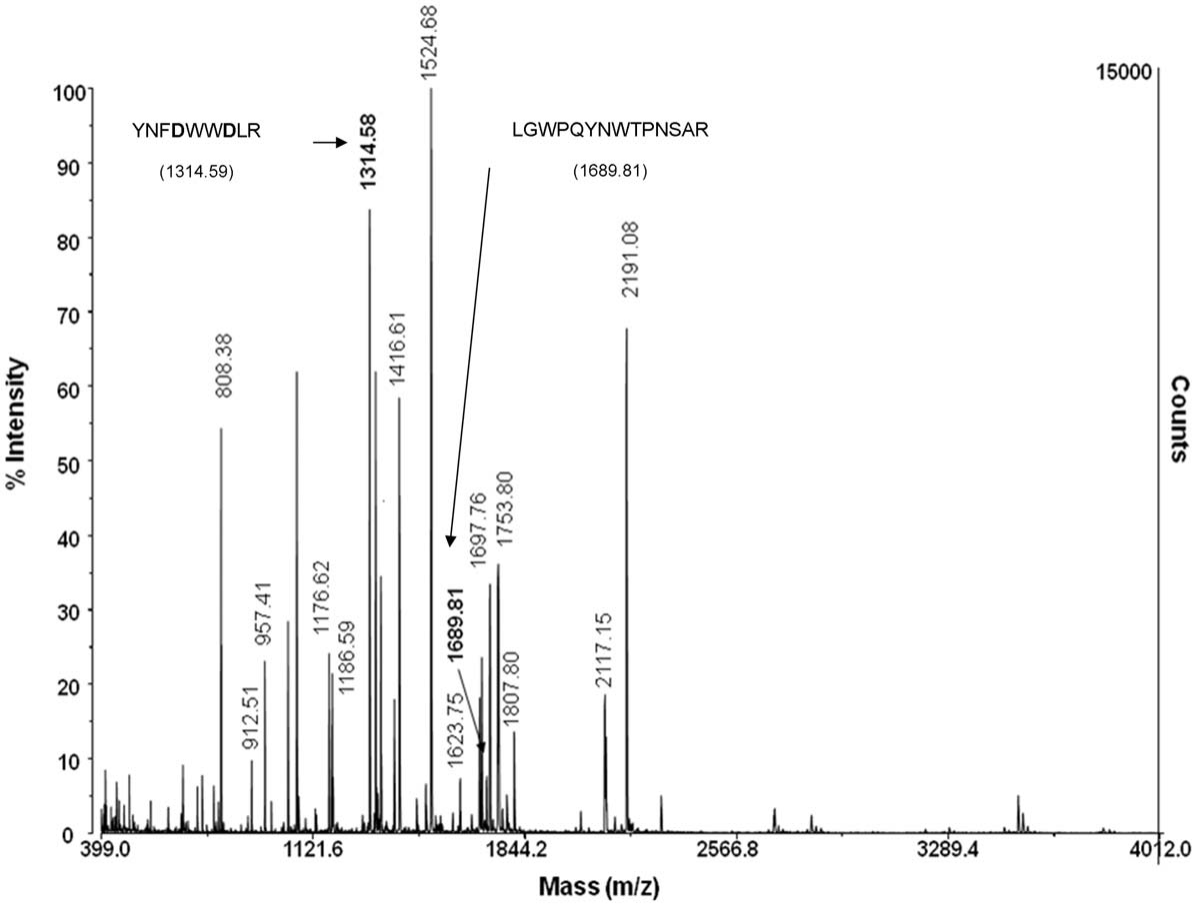
MALDI TOF/TOF spectrum of mutant (Y465D) ACE after tryptic digest. An in-gel tryptic digest was performed on mutant (Y465D) ACE, the cysteines were protected using iodoacetamide, and the total digest subjected to MALDI TOF/TOF using the matrix α-cyano-4-hydroxycinmanic acid. Masses corresponding to predicted ACE peptides are labeled. The masses corresponding to peptide containing Y465D and the C-terminal cleavage peptide are indicated in bold.

A possible explanation for the striking increase in shedding observed for ACE-Y465D as well as different effects of known inducers and inhibitors of ACE shedding is that the mutation has altered the interaction of the secretase with ACE causing aberrant shedding, most likely at a novel cleavage site. To test this hypothesis, the cleavage site of ACE-Y465D was determined by MALDI MS/MS (Fig. 7, Table 1). A tryptic digest of purified soluble ACE revealed a fragment (*m/z* 1689.81, expected *m/z* 1689.81), which corresponds to the C-terminal cleaved peptide (L1190-R1203). The peptide, containing Y465D (Y459-R467) was identified as an *m/z* 1314.58 fragment (expected *m/z* 1314.59), confirming the presence of the mutation in the protein sequence. From these results, it seems that the Y465D transition does not affect the cleavage site of the ACE sheddase(s) and that cleavage occurs by the same mechanism as WT ACE.

**Table 1:** MALDI MS/MS analysis of sACE-Y465D after tryptic digest.

Residue no	Expected mass [MH]^+^	Observed mass [MH]^+^	Peptide sequence identified by MS/MS
53–71	2190.70	2191.08	
133–151	2121.03	2121.04	
188–199	1416.61	1416.61	
327–340	1753.76	1753.80	
433–446	1724.92	1724.91	
447–453	808.41	808.38	
**459–467**	**1314.59**	**1314.58**	**YNFDWWDLR**
518–532	1807.81	1807.80	
623–629	957.43	957.41	
751–762	1623.76	1623.75	
776–785	1176.64	1176.62	
798–811	1697.77	1697.76	
812–828	2117.15	2117.15	
1055–1065	1524.69	1524.68	
1068–1077	1186.60	1186.59	
1174–1180	912.53	912.51	
**1190–1203**	**1689.81**	**1689.81**	**LGWPQYNWTPNSAR**

Masses (*m/z*) corresponding to ACE peptides, the peptide containing Y465D (*underlined*) and the C-terminal peptide are indicated in *bold*.

### *In silico* analysis of the Y465D mutation in ACE

Our results convincingly demonstrate that a single amino-acid substitution in the N domain of ACE leads to significantly increased ACE shedding as the result of gross conformational changes of the two domains of sACE, but do not explain the fine mechanisms of how this may occur. Previously we demonstrated that ∼15% of sACE expressed on the surface of CHO cells exists as dimers [39], which was later confirmed for porcine and human endothelial cells [59]. Moreover purified sACE as well as truncated N domain, but not C domain, were able to form dimers in reverse micelles [39]. Furthermore, analysis of the effect of mAbs on the induction and inhibition of ACE shedding [60] and on ACE dimerization in reverse micelles suggests a link between ACE dimerization and shedding [39]–[40].

**Figure 8:**
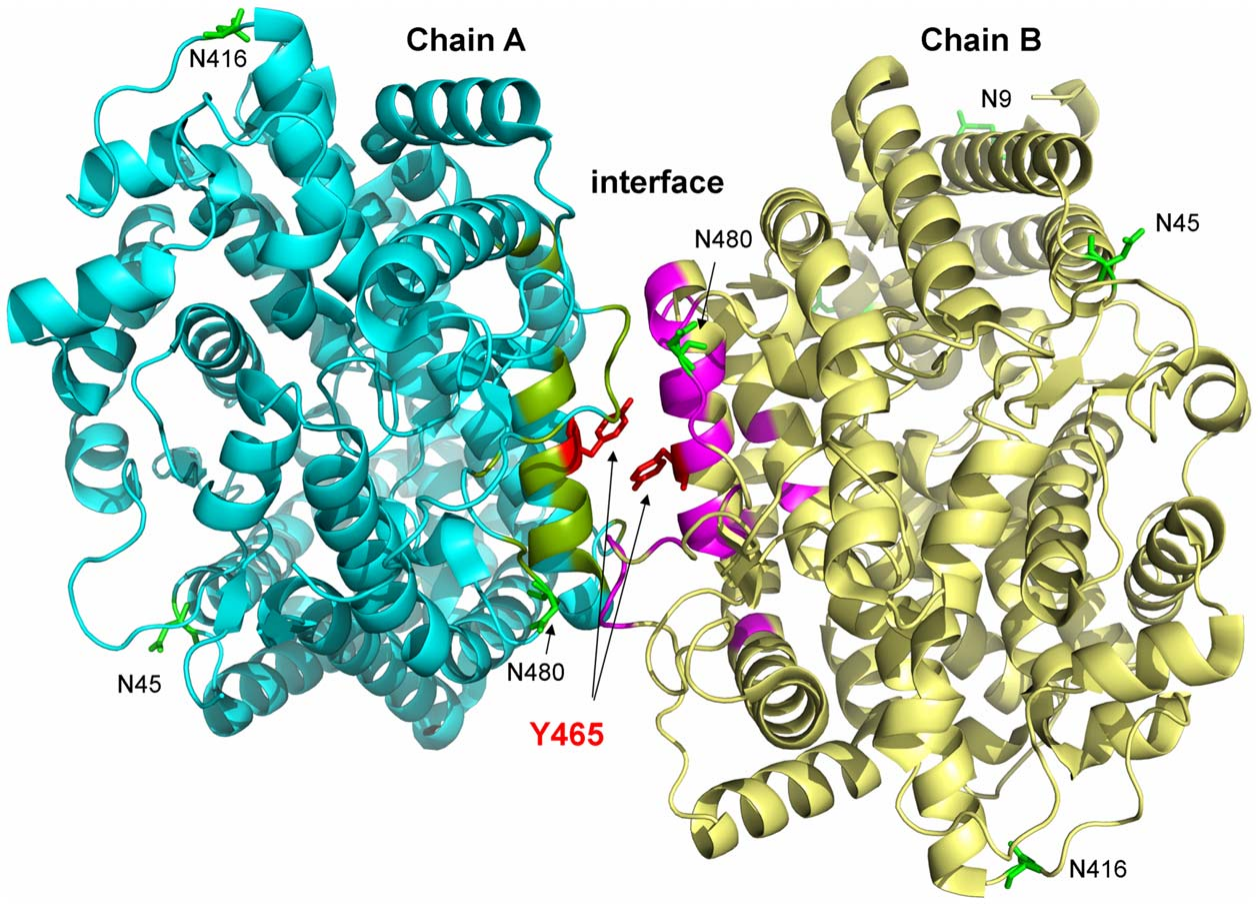
Dimer of N domain of ACE. The dimer of the N domain was shown based on the structure of the N domain with N-domain specific inhibitor RXP407 [38] - PDB accession # 3NXQ. The interface between monomers A (cyan) and B (yellow) is highlighted in green and magenta. Y465 is highlighted in red. N9, N45, N416, and N480 (glycosylation sites) are rendered in green.

**Figure 9:**
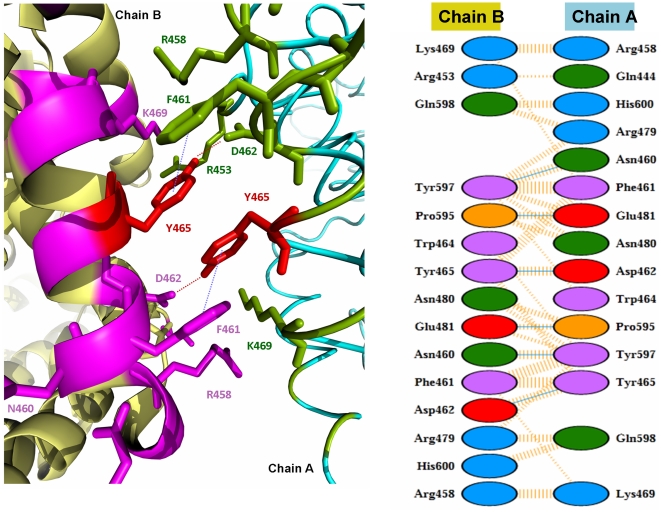
Dimer interactions observed at the interface of N domain. **A.** X-ray model showing all the residues at the interface. The color scheme is the same as in Fig. 8. Hydrogen bonds and non-covalent interactions between Y465 (red) and other residues are shown by dashed lines. **B.** A schematic representation of the protein-protein interactions at the dimer interface identified by PDBSum [http://www.ebi.ac.uk/pdbsum/]. The residues and their interactions are colored using the following notation: hydrogen bonds –blue+dashed lines, non-bonded contacts - dashed lines, positive residues - blue, negative residues - red, neutral residues - green, proline residues - orange, aromatic residues – magenta. Y465 is involved in a hydrogen bond with D462 and hydrophobic non-bonded interaction with F461.

The crystal structure of the N domain resolved as two molecules per asymmetric unit, interacting via an interface encompassing helices α10 and α27 on both molecules (2C6N) [64]. Recently, a minimally glycosylated N domain (Ndom389) co-crystallized with the domain selective phosphinic inhibitor, RXP407, (3NXQ), demonstrated a similar protein-protein interface involving helices α10, α20 and α21 [38] where the two molecules associate as mirror images with a C2 axis of symmetry. Analysis of the former structure demonstrates the localization of Y465 on helix α21 of both molecules within this interface (Figure 8). The similarity of these interfaces in both crystal structures is suggestive of their involvement in protein-protein interactions, such as dimerization, *in vivo*. The most likely interface for dimerization of the N domain and potential residues involved were identified using the protein interfaces, surfaces and assemblies service (PISA) program [56] and PDBsum [http://www.ebi.ac.uk/pdbsum/] and given in Table 2 and 3. According to this analysis, dimer association is driven mostly by hydrogen bonds and non-bonded contacts at the interface formed by helix α21 (nomenclature as in [64]) of both molecules (Figure 9). Y465 forms a hydrogen bond with D462 and a non-covalent interaction with the phenyl ring of F461. A computational model suggests that substitution of Tyr465 with an Asp results in a subsequent loss of hydrogen bonding and non-covalent interactions with F461 (Table 2). Thus, the loss of energetically important interactions in the interface may cause conformational changes that alter dimer formation, subsequently resulting in changes in ACE shedding, such as those observed in this study.

**Table 2:** Contact residues involved in hydrogen bonding at the interface of native and mutated ACE based on PISA analysis.

Native protein			Single Mutation in chain A			Double Mutation in chains A and B		
Residue	Distance[Å]	Residue	Residue	Distance[Å]	Residue	Residue	Distance[Å]	Residue
B:**TYR 465[OH]**	**2.65**	A:**ASP 462[OD1]**	B:GLN 444[OE1]	3.27	A:ARG 453[NH2]	B:GLN 444[OE1]	3.27	A:ARG 453[NH2]
B:GLN 598[NE2]	3.41	A:ARG 479[O]	B:TYR 597[OH]	3.12	A:ASN 460 [ND2]	B:TYR 597[OH]	3.12	A:ASN 460 [ND2]
B:GLU 481[N]	2.93	A:PRO 595[O]	B:PRO 595[O]	2.99	A:GLU 481[N]	B:PRO 595[O]	2.99	A:GLU 481[N]
B:ASN 460[ND2]	3.12	A:TYR 597[OH]	B:ARG 479[O]	3.45	A:GLN 598[NE2]	B:ARG 479[O]	3.45	A:GLN 598[NE2]
B:GLN 444[0E1]	3.27	A:ARG 453[NH2]	B:GLN 598[OE1]	3.25	A:HIS 600[NE2]	B:GLN 598[OE1]	3.25	A:HIS 600[NE2]
B:TYR 597[OH]	3.12	A:ASN 460[ND2]	B:**TYR 465[OH]**	**2.65**	A:**ASP 462[OD1]**	B:GLN 598[NE2]	3.41	A:ARG 479[O]
B:**ASP 462[OD1]**	**2.55**	A:**TYR 465[OH]**	B:GLN 598[NE2]	3.41	A:ARG 479[O]	B:GLU 481[N]	2.93	A:PRO 595[O]
B:PRO 595[O]	2.99	A:GLU 481[N]	B:GLU 481[N]	2.93	A:PRO 595[O]	B:ASN 460[ND2]	3.12	A:TYR 597[OH]
B:ARG 479[O]	3.45	A:GLN 598[NE2]	B:ASN 460[ND2]	3.12	A:TYR 597[OH]			
B:GLN 598[OE1]	3.25	A:HIS 600[NE2]						

The residue column gives the description of the chain, residue name, residue number and atom involved in hydrogen bonding. The distance column represents the calculated distance between the two interacting atoms. Bonding with Y465 is marked by bold. It was observed that transition from the native to the single followed by the double mutation results in systematic loss of H-bonding at the interface.

**Table 3:** Solvent accessible surface area [Å^2^] of the interface for each chain of N domains.

Native protein		Single Mutation in chain A		Double Mutation in chains A and B	
Chain A	Chain B	Chain A	Chain B	Chain A	Chain B
933.9	932.6	915.9	920	904.4	897.8

Y465D mutation in chain B results in steady decrease of solvent accessible surface area at the interface.

**Figure 10:**
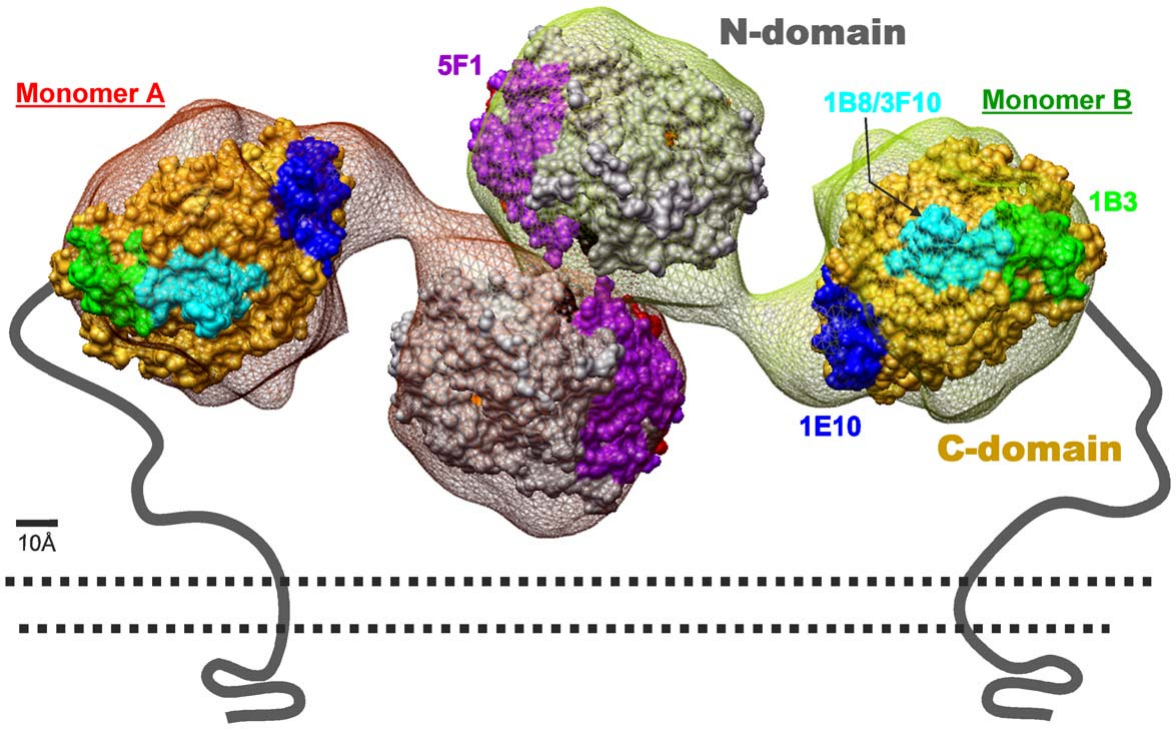
A putative model of an ACE dimer attached to cell membrane. The yellow and red “dumbbells” correspond to the EM model of porcine ACE. ACE monomers **A** (red) and **B** (yellow) are oriented such that they form a “back-to-back” complex. The N domain is rendered grey, C domain – beige.

In a model of an ACE homodimer attached to the cell membrane and marked with the key epitopes, the monomers are oriented as mirror images associating via the N domains, with the C domain of each monomer accessible for interactions with the cell membrane (Fig. 10). This model reflects the conformational fingerprinting of sACE-Y465D where binding of mAbs to at least three epitopes was affected, namely 5F1, 1G12/6A12, and 1E10. In addition, if dimerization requires a gross change in conformation to allow for interaction of the N domains of each monomer, the conformation of the terminal region of the C domain, including epitopes 1B8/3F10 and 1B3, and the juxtamembrane stalk region, beyond P1199 would be affected. Moreover, the epitope of mAb 5F1 appears to be significantly masked by dimer formation via the N domain which could explain the dramatic decrease in 5F1 binding with both cell-associated and soluble ACE-Y465D (Fig. 3) and mutant ACE expressed on the cell surface (Fig. 4). Comparison of these observations with the conformational fingerprint of mutant ACE (Fig. 3) and the results of cell ELISA (Fig. 4) shows that the theoretical and experimental results mostly agree, rendering the proposed dimer an acceptable working model for further investigation.

### Conclusions

In this study, we ascribed a 5–7-fold elevation of plasma ACE to a novel ACE mutation in which the codon GAT, encoding Tyr465 in the N domain of ACE, is substituted in one allele by TAT coding for Asp. The identification and characterization of this and other [32], [33] mutations that result in increased shedding of ACE are of considerable clinical importance considering that a genetically determined increase in plasma ACE may lead to false diagnosis of sarcoidosis and, consequently, to unnecessary long-term immunosuppressive treatment [34]. Levels of plasma ACE appear to be affected in two ways: either through the regulation of gene expression and genetically linked to loci in the promoter region of the ACE gene and other genomic loci, or through spontaneous alterations of the ACE gene itself as in the case of the Tyr465Asp and Pro1199Leu mutations. The link between the alteration in plasma ACE levels due to the Y465D mutation and the symptoms of nausea, vomiting, pain, depression and/or fatigue in affected individuals remains unclear. Particularly since this is the first mutation described that is associated with adverse clinical symptoms.

The main reason for the absence of clinical abnormalities in these patients is that their tissue ACE, generally 10- to 30-fold higher than blood ACE, remains unaltered and thus the overall substrate hydrolysis does not change significantly. We hypothesized that the reason why most members of this family with the Y465D mutation (6 out of 7) have one or more of the above symptoms is due to elevation of plasma substance P or other neuropeptides shown to be involved in similar neuropathophysiological disorders [Nesterovitch et al. 2011, in preparation]. There are two possible mechanistic scenarios: firstly, symptomatic family members could have an additional mutation in another gene which is responsible for aberrant metabolism and/or processing of neuropeptides. Secondly, the Y465D mutation could result in a second phenotype - altered hydrolysis of ACE substrates, such as substance P or similar and as yet unknown neuropeptides. We demonstrated the rate of hydrolysis of artificial, synthetic substrates Hip-His-Leu and Z-Phe-His-Leu was not changed due to the Y465D substitution. However, these experiments were carried out with soluble ACE proteolytically released from the membrane and the conformation and kinetics of the soluble and membrane-bound forms of ACE can differ significantly [61]–[62]. Thus, one cannot exclude the likelihood that one mutation of ACE causes both elevated blood ACE due to increased ACE shedding, and elevated levels of neuropeptide(s), such as substance P, resulting in neuropathological symptoms.

In summary, we have identified a novel Y465D mutation that results in dramatic elevation of serum ACE, and is associated with adverse clinical symptoms. Since elevated plasma ACE is often taken as a marker of disease activity such as in sarcoidosis and Gaucher's disease, it is important for clinicians and medical scientists to be aware of the alternative genetic causes of elevated blood ACE that may or may not be linked to disease.
